# Critical analysis of the major and ancillary imaging features of LI-RADS on 127 proven HCCs evaluated with functional and morphological MRI: Lights and shadows

**DOI:** 10.18632/oncotarget.17227

**Published:** 2017-04-19

**Authors:** Vincenza Granata, Roberta Fusco, Antonio Avallone, Francesco Filice, Fabiana Tatangelo, Mauro Piccirillo, Roberto Grassi, Francesco Izzo, Antonella Petrillo

**Affiliations:** ^1^ Department of Radiology, “Istituto Nazionale Tumori, IRCCS, Fondazione G. Pascale”, Naples, Italy; ^2^ Department of Abdominal Oncology, “Istituto Nazionale Tumori, IRCCS, Fondazione G. Pascale”, Naples, Italy; ^3^ Department of Diagnostic Pathology, “Istituto Nazionale Tumori, IRCCS, Fondazione G. Pascale”, Naples, Italy; ^4^ Department of Abdominal Surgical Oncology, Hepatobiliary Unit, “Istituto Nazionale Tumori, IRCCS, Fondazione G. Pascale”, Naples, Italy; ^5^ Department of Radiology, Second University of Naples, Piazza Miraglia, Naples, Italy

**Keywords:** HCC, Li-RADS, magnetic resonance imaging, diffusion weighted imaging, dynamic contrast assessment

## Abstract

**Purpose:**

To report a critical analysis of major and ancillary MR imaging features in assessment of HCC.

**Methods:**

Retrospectively we evaluated 70 cirrhotic patients with 173 nodules, which were subjected to MR study at 0 time (MR0), after 3 (MR3) and 6 months (MR6) using two different contrast media. EOB-GD-DTPA was injected at MR0 and MR6, while Gd-BT-DO3A at MR3. Three expert hepatic radiologists reviewed all images, recording, according to LI-RADS, the size, the presence and quality of arterial-phase hyperenhancement, washout and capsule appearance, threshold growth. Additionally, we recorded signal intensity (SI) on T2-W images, on DWI, on apparent diffusion coefficient (ADC) maps and SI on T1-W images of EOB-GD-BPTA hepatospecific phase. Median value of ADC and of Intravoxel incoherent motion related parameters were assessed.

**Results:**

127 HCCs and 24 dysplastic nodules were assessed. Hypervascular on arterial phase was found in 84 HCCs, washout appearance in 124, capsule appearance in 111, hypointensity on hepatospecific phase in 127, hyperintensity on T2-W sequences and restricted diffusion in 107. Hyper vascular on arterial phase was found in 17 dysplastic nodules, wash-out appearance in 2, hypointensity on hepatospecific phase in 7 while no dysplastic nodules showed capsule appearance, hyperintensity on T2-W and restricted diffusion. Highest accuracy was obtained by washout appearance and hypointense signal on hepatospecific phase (97% and 95%).

**Conclusions:**

Hypointensity on hepatospecific phase and washout appearance are the most relevant diagnostic sign for differentiating low-risk from high-risk HCC nodules. The capsule appearance, T2-W hyperintensity and restricted diffusion have high positive predictive value.

## INTRODUCTION

Liver Imaging Reporting and Data System (LI-RADS) is a score to report and interpret hepatic imaging characteristics on computed tomography (CT) and magnetic resonance (MR) studies in patients with risk for hepatocellular carcinoma (HCC) [1–2]. European Association for the Study of the Liver (EASL), in accordance with the guidelines of the American Association for the Study Liver Diseases (AASLD), recommended that in order to characterize HCC, non-invasive criteria can exclusively be applied to cirrhotic patients using 4-phase multidetector CT scan or dynamic contrast-enhanced MR examination [3]. Diagnosis is based on the identification of the HCC typical mark (hypervascular in the arterial phase and washout in the portal venous or delayed phases) [3]. Considering that the imaging characteristics used to identify hepatic lesions, could lead to uncertainty in characterization and absence of reproducibility both in clinical care and in research [4], American College of Radiology (ACR) sustained the development of LI-RADS in order to standardize the interpreting, reporting and data collection of HCC imaging. LI-RADS system meets the necessity to perform an exact definition of HCC, in fact it is clinically significant to discriminate between HCC and other malignancies such as cholangiocarcinoma or benign nodules, because the management changes substantially [1]. Moreover, current systems neglect the criteria for vascular invasion diagnosis by HCC, which has important implications in staging and treatment [2]. In the current (v. 2014) LI-RADS [5], the HCC diagnosis was done primarily on the presence/absence of major imaging features. Major imaging features are used to categorize LR-3, LR-4, and LR-5; these comprise arterial-phase hyperenhancement, tumor diameter, washout appearance, capsule appearance and threshold growth [5]. Ancillary imaging features could be employed to change the LI-RADS category [1, 5]. Ancillary features giving preferentiality to malignancy (diffusion restriction, moderate T2 hyperintensity, T1 hypointensity on hapatospecifc phase) could be used to upgrade system by one or more categories, but not beyond LR-4 [1, 5–8]. In contrast, ancillary features favoring benignity can be used to decrease category down to LR-1 [5]. Our purpose is to report a critical analysis of MR Imaging major and ancillary features to assess HCC smaller than 20 mm.

## RESULTS

Among 173 nodules, eight were combined hepatocellular-cholangiocarcinoma, seven metastases, 7 cirrhotic regenerative nodules, 24 dysplastic nodules and 127 HCCs (median 1.8 lesions for patients). All HCCs were histologically classified according to the major Edmondson-Steiner grade on final pathologic reports as follows: grade 1 (n = 30), grade 2 (n = 61), grade 3 (n = 36), and grade 4 (n = 0).

We does not evaluated the imaging features of combined nodules, metastases and cirrhotic regenerative nodules while we analyzed HCCs and dysplastic nodules.

We analyzed only lesions with a diameter between 12 and 20 mm.

**Figure 1:**
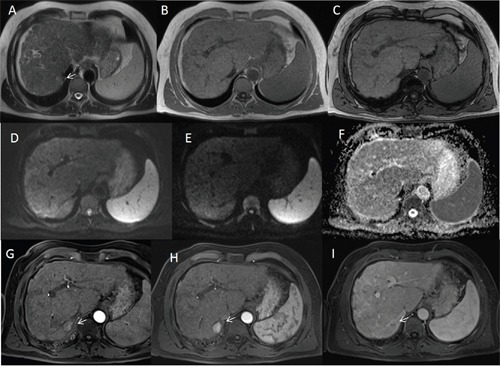
Man sixty-five years old with HCC on VII hepatic segment The lesion appears hyperintense (arrow) on HASTE T2-W axial plane image sequence **A** while on in-of-phase T1-W and out of phase T1-W sequences **(B** and **C)** the lesion is isointense. On DW images **(D** b0 s/mm^2^ DW image, **E** b800 s/mm^2^ DW image, **F** ADC map) the HCC shows isointense signal. During arterial phase **(G, H** and **I)** the HCC is hypervascular (arrow) with wash-out in portal phase (arrow).

When we evaluated T2-W images, arterial phase, portal phase and hepatospecific phase of contrast study we found difference of measured lesion size on different sequences, but this is not statistically significant (differences median value is 0.2 mm, range 0.1-0.3 mm; p value > 0.05 at Kruskal Wallis test). So we reported a median value of lesion size measured by 4 sequences that was 18 mm (range 12- 20 mm) (Figure 1).

**Figure 2:**
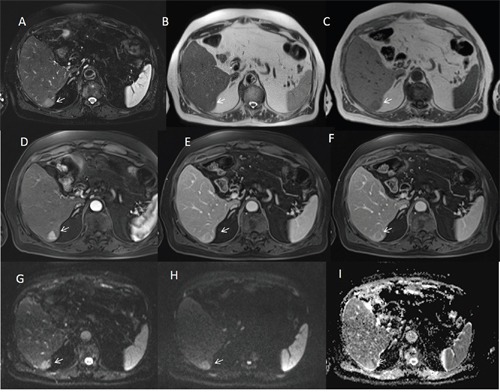
Man seventy-three years old with typical HCC on VI hepatic segment The HCC is hyperintense (arrow) on T2-W sequences **(A** and **B)**, hypointense (arrow) on T1-W **(C)** sequences, hyper vascular (arrow) on arterial phase **(D)**, with wash-out appearance (arrow) on portal phase **(E)** and capsule appearance (arrow) on equilibrium phase **(F)** of contrast study with Gd-BT-DO3A. The HCC shows (arrows) restrict diffusion **(G** b50 s/mm^2^ DW image, **H** b800 s/mm^2^ DW image, **I** ADC map).

**Figure 3:**
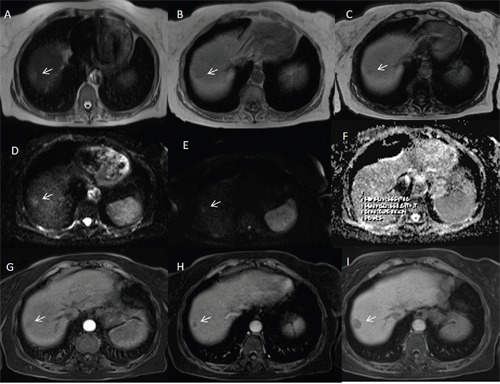
Woman fifty-three years old with atypical HCC on VII-VIII hepatic segment The HCC is hyperintense (arrow) on T2-W sequences **(A)** and hypointense (arrow) on T1-W in-of- phase and out-of-phase sequences **(B** and **C** out-of-phase). The lesion shows (arrows) restrict diffusion **(D** b50 s/mm^2^ DW image, **E** b800 s/mm^2^ DW image, **F** ADC map). During arterial phase **(G)**, it is not hypervascular (arrow) appearance, while there is wash-out appearance (arrow) on portal phase **(H)** and capsule appearance (arrow) on equilibrium phase **(I)** of contrast study with Gd-BT-DO3A.

84 HCCs (13 grade 1, 42 grade 2 and 29 grade 3) had typical hallmark (hypervascular in the arterial phase with washout in the portal and equlibrium phases) (Figure 2); 40 (15 grade 1, 18 grade 2 and 7 grade 3) nodules were no hypervascular in arterial phase, but had washout appearance (atypical nodules) (Figure 3). We found the capsule appearance in 111 (89,5%) nodules.

Additionally, we detected 15 nodules only during hepatospecific phase. Among them 3 (20%) were HCCs (2 grade 1 and 1 grade 2), 5 (33,3%) dysplastic nodules and 7 (46,7%) cirrhotic regenerative nodules.

**Figure 4:**
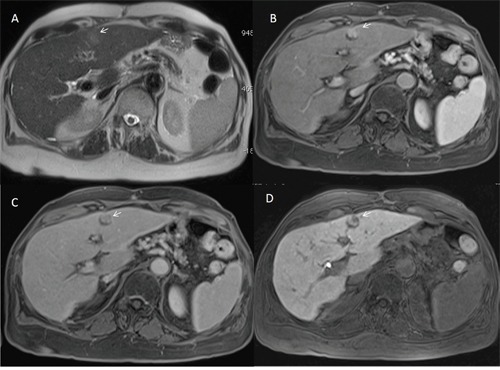
Man sixty-one years old with nodule of HCC in dysplastic nodule on II hepatic segment The nodule is hyperintense (arrow) on T2-W sequences **(A)**, hyper vascular (arrow) on arterial phase **(B)**, isointense on portal phase **(C)** with a peripheral hypointense signal (arrow) on hepatospecific phase **(D)** of contrast study with EOB-GD-DTPA.

All 127 HCCs were hypointense on hepatospecific phase (Figure 4).

**Figure 5:**
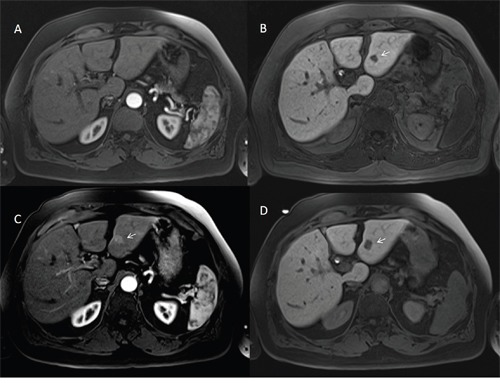
Man seventy-four years old with HCC on III hepatic segment At MR0 study with EOB-GD-DTPA the lesion is not hypervascular on arterial phase **(A)** and it is evident on hepatospecific phase **(B)** of contrast study (arrow). At MR6 study with EOB-GD-DTPA, the HCC is hypervascular (arrow) on arterial phase **(C)** with hypointense signal (arrow) on hepatospecific phase **(D)** of contrast study.

Among the 3 HCC detected by hepatospecific phase, one (grade 2) at MR6 became hypervascular on arterial phase (Figure 5) with wash-out on portal phase. The signal was isointense on T2-W and DWI.

No nodule grew during follow up.

All typical nodules were hyperintense on T2-W and DWI, with diffusion restriction and hypointense signal on ADC maps, 23 (57,5 %) out of atypical nodules were hyperintense on T2-W and DWI, with diffusion restriction and hypointense signal on ADC maps, while 17 (42,5%) were iso-hypointense on T2-W and DWI and isointense on ADC maps. The median value for ADC was 1,47 x10^-3^ mm^2^/s (range 0,94-2,44 x10^-3^ mm^2^/s), for fp was 33,3% (range 12,14-54,08%), for Dp 45,3 x10^-3^ mm^2^/s (range 13,7-52,7 x10^-3^ mm^2^/s) and for Dt 0.9×10^-3^ mm^2^/s (range 0.81-1,51 x10^-3^ mm^2^/s). Among typical and atypical nodules we found an overlapping of ADC and IVIM derived parameters values. We not found difference significant statistically between typical and atypical nodules using ADC or IVIM parameters values (p value < 0.05 at Kruskal Wallis test).

**Figure 6:**
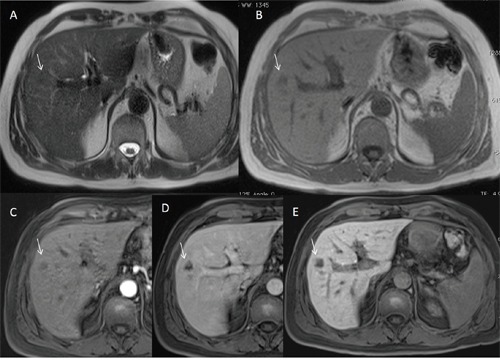
Man fifty-five years old with dysplastic nodule on V hepatic segment The nodule is isointense (arrow) on T2-W sequence **(A)** and hypointense (arrow) on T1-W sequence **(B)**. The lesion is hypointense on arterial **(C)**, portal **(D)** and hepatospecific **(E)** phase of contrast study with EOB-GD-DTPA.

Among 24 dysplastic nodules, 5 (20,8 %) were detected only during hepatospecific phase. Seventeen lesions (70,8%) were isointense on T2-W, DW and ADC map with hyperenhancement during arterial phase and isointense signal during portal, equilibrium and hepatospecific phase of contrast studies. Two lesions (8,3%) were isointense on T2-W, DW, ADC map and hypointense on arterial, portal and hepatospecific phase of contrast studies (Figure 6).

**Figure 7:**
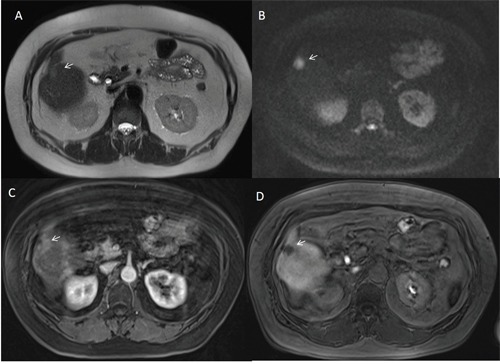
Woman seventy-three years old with HCC on VI hepatic segment The HCC is hyperintense (arrow) on T2-W sequence **(A)** with restrict diffusion (arrow) and hyperintense signal on b800 s/mm^2^ DW image **(B)**. During arterial phase of contrast study **(C)** with EOB-GD-DTPA, the degree of image quality degradation caused by pulsatile blood flow ghost is severe (arrow). During hepatospecific phase **(D)** of contrast study the HCC is hypointense (arrow).

We evaluated 210 arterial phases (70 at MR0, 70 at MR3 and 70 at MR6) to assess the image quality degradation. The median score for all arterial phase with Gd-BT-DO3A (70 studies) was 1, while the median score for all arterial phase with GD-EOB-DTPA (140 studies) was 3 (range 1-4); there was significant statistically difference between the quality on arterial phase with Gd-BT-DO3A and the quality on arterial phase with GD-EOB-DTPA (p value = 0.03 at Kruskal Wallis test). In 25 (17,8 %) cases the images, during arterial phase with GD-EOB-DTPA, were uninterpretable (Figure 7).

When we analyzed the degree of hyperenhancement of arterial phase, we found that the degree was higher with Gd-BT-DO3A (a median value of 4 comparet to the median value of 2.6 with GD-EOB-DTPA, and we found no significant statistical differences among median values at MR0 and MR6 (equal to 2.6) using GD-EOB-DTPA; there was significant statistically difference between the degree of hyperenhancement of arterial phase with Gd-BT-DO3A and the degree of hyperenhancement of arterial phase with GD-EOB-DTPA (p value =0.02 at Kruskal Wallis test).

Our results are summarized in Table 1 and 2.

**Table 1:** HCC Nodules Imaging Features

Description	Numbers (%)
**127 HCCs**	30 (23,6%) grade 1, 61 (48,03%) grade 2, 36 (28,3%) grade 3 and 0 (0%) grade 4
Size	18 mm (range 12- 20 mm)
Hyper vascular on arterial phase	84 (66,1%)
Wash-out appearance	124 (97,6 %)
Capsule appearance	111 (87,4%)
Hypointense Signal on Hepatospecific phase of contrast study	127 (100%)
Hyperintense Signal on T2-W sequences	107 (84,2%)
Restricted Diffusion	107 (84,2%)
ADC	Median value: 1,47 x10^-3^ mm^2^/s (range 0,94-2,44 x10^-3^ mm^2^/s)
fp	Median value: 33,3 % (range 12,14-54,08 %)
Dp	Median value: 45,3 x10^-3^ mm^2^/s (range 13,7-52,7 x10^-3^ mm^2^/s)
Dt	Median value: 0.9×10^-3^ mm^2^/s (range 0.81-1,51 x10^-3^ mm^2^/s)
**Quality of arterial phases**	The median score for all arterial phase with Gd-BT-DO3A (70 studies) was 1
	The median score for all arterial phase with GD-EOB-DTPA (140 studies) was 3 (range 1-4)In 25 (17,8 %) cases the images were uninterpretable
**Degree of hyperenhancement of arterial phase**	The median value was 4 with Gd-BT-DO3A
	The median value was 2.6 with GD-EOB-DTPA

**Table 2:** Dysplastic Nodules Imaging Features

Description	Numbers (%)
**24 dysplastic nodules**	
Size	18 mm (range 12- 20 mm)
Hyper vascular on arterial phase	17 (70,8%)
Wash-out appearance	2 (8,3%)
Capsule appearance	0 (0%)
Hypointense Signal on Hepatospecific phase of contrast study	7 (29,16%)
Hyperintense Signal on T2-W sequences	0 (0%)
Restricted Diffusion	0 (0%)
**Degree of hyperenhancement of arterial phase**	The median value was 4 with Gd-BT-DO3A
	The median value was 2.6 with GD-EOB-DTPA

Table 3 reports the diagnostic accuracy for each MR imaging features. Highest accuracy was obtained by washout appearance and hypointense signal on hepatospecific phase of contrast study (97% and 95%, respectively).

**Table 3:** Diagnostic Accuracy of Imaging Features

	Sensitivity	Specificity	PPV	NPV	Accuracy	p value of Fisher's exact test
Hyper vascular on arterial phase	66,14	29,17	83,17	14,00	60,26	0.8140
Wash-out appearance	97,64	91,67	98,41	88,00	96,69	0.0001
Capsule appearance	87,40	100,00	100,00	60,00	89,40	0.0001
Hypointense Signal on Hepatospecific phase of contrast study	100,00	70,83	94,78	100,00	95,36	0.0001
Hyperintense Signal on T2-W sequences	84,25	100,00	100,00	54,55	86,75	0.0001
Restricted Diffusion	84,25	100,00	100,00	54,55	86,75	0.0001

Abbreviations: PPV = positive predictive value; NPV = negative predictive value.

## DISCUSSION

In the management of patients with HCC is crucial its early diagnosis [9–13]. The identification of the vascular profile (contrast uptake in the arterial phase followed by washout in the venous phases) has permitted the non-invasive diagnostic criteria definition for HCC according to AASLD and EASL-EORTC guidelines. If this vascular profile is identified on dynamic CT or MR Imaging using extracellular contrast media in patients at high risk of HCC, the diagnosis is established with the 100% of specificity [14]. However, these criteria have a sensitivity range of 50–60% in nodules smaller than 20 mm; therefore a biopsy is still needed [15]. In this study we analyzed only nodules smaller than 20 mm, however there is a consideration should be make. Considering that the tumor diameter is defined as the largest dimension, measured in the imaging sequence, phase, and plane in which the margins are most sharply defined, we found a difference among the lesion diameter when we analyzed different sequences, with higher difference among arterial, portal and hepatospecific phase and, although it is not significant statistically, the question born when the HCC is 10 mm or 20 mm, to the bounds of the range. Moreover, the possibility to find foci of HCC in dysplastic nodules, as we established in this study, opens the question of how to measure the HCC (the entire nodule or willful part). We considered a mean value obtained from to each sequence, however it is could be considered a limit. Therefore, how and in which sequence to use should be standardized in order to measure the nodules according to LI-RADS, considering there are two or three categories according to the size criteria (<20 mm versus >20 mm for hypovascular observations and <10 mm versus 10-20 mm versus >20 mm for hypervascular observations) [1–2]. According to our data we think that the best diagnostic performance may be obtained on hepatospecific phase when it is employed EOB-GD-DTPA, if there is not a strongly parenchymal distortion.

Arterial phase hyperenhancement is a crucial precondition to define HCC (LR-5) [1–2]. However, it is non-specific condition and may be detected in benign pathologies such as dysplastic nodules and arterioportal shunts. In our series we found hyperenhancement on 17 dysplastic nodules and on 84 HCCs; hyperenhancement had a sensitivity of 66,14 %, a specificity of 29,17%, a positive predictive value of 83,17%, a negative predictive value of 14,0%, a diagnostic accuracy of 60,26% (p value of 0.8140). Holland et al [16] demonstrated, in patients with HCC, that the majority (93%) of hypervascular lesions on arterial phase that were occult on T2-weighted image and portal and/or equilibrium phase were non neoplastic. Conversely, Kim et al [17] demonstrated that the most significant findings associated with HCC, in nodules smaller than 20 mm, were arterial phase hyperintensity. In our series we found that 43 HCCs were no hypervascular in arterial phase, therefore, we think that arterial phase hyperenhancement is a prerequisite but not sufficient for LR-5 classification. Another open question is which contrast medium should be used. In fact when we analyzed the degree of arterial phase hyperenhancement, we found that the degree was higher with Gd-BT-DO3A than GD-EOB-DTPA, with significant statistically difference (p value = 0.02 at Kruskal Wallis test). Also, the image quality degradation was lower with Gd-BT-DO3A (median score was 1) than with GD-EOB-DTPA (median score was 3). There was significant statistically difference between the quality on arterial phase with Gd-BT-DO3A and the quality on arterial phase with GD-EOB-DTPA (p value = 0.03 at Kruskal Wallis test) and in 25 cases the images, during arterial phase with GD-EOB-DTPA, were uninterpretable. These results are worthy of some considerations. First GD-EOB-DTPA is a liver-specific agent, taken up by hepatocytes [18]. It can be injected as an intravenous bolus, providing data about lesion vascularity in the different phases of contrast circulation. Additionally functional data can be obtained in the delayed, hepatobiliary phase [18]. Conversely, Gd-BT-DO3A is a non liver-specific agent and it provides data only about lesion vascularity, although with a better quality of arterial phase and a better hyperenhancement. Second our results showed that the hyperenhancement during arterial phase has a lower sensitivity, specificity, positive predictive value, negative predictive value and diagnostic accuracy than hypointensity on hepatospecific phase. Third HCC is the evolution of cirrhosis, so that when we evaluate a nodule we assessing all hepatic parenchyma in which we can found nodules in different phase of evolution or treated nodules. Considering that ablated area are evaluated according to mRECIST [19], in HCC patient we should obtain the best quality of arterial phase. In this setting we suggest to evaluate the HCC patients alternating these contrast media or considering clinic indication [10, 13, 20–21]. Washout is defined as temporal contrast-enhancement reduction relative to liver from an earlier to a later phase resulting in hypoenhancement in portal or delayed phase [22–23]. This may reveal several phenomena: rapid venous drainage, reduced portal venous supply and background liver later enhancement particularly with hepatobiliary agents [24]. Some researchers reported a variation in the timing of washout in the portal venous and delayed phases [25]. In a pilot study on enhancement of 112 histologically proven HCCs, arterial phase hypervascularity was on 74 (77, 96%) and portal washout within 90 seconds on 72 (74, 97%) in the majority of moderately differentiated HCC. However, the authors found that well-differentiated and poorly differentiated HCCs had an atypical enhancement patterns where 25 of 97 (26%) showed washout between 91 and 180 seconds and 21 of 97 (22%) showed late washout between 180 and 300 seconds [25]. In our series washout appearance was found on 97,6 % of HCCs with sensitivity of 97,64%, specificity of 91,67%, PPV of 98,41% and NPV of 88,00% and the highest diagnostic accuracy (96,69%) compared to other imaging feature. Our results confirmed the data by Becker et al [26], that showed as the diameter and washout criteria using a step wise LI-RADS decision tree for LR3-5 observations allowed faster categorization with better inter-observer reliability while maintaining the excellent diagnostic accuracy of the most recent LI-RADS v2014. Choi et al [27] demonstrated as HCCs smaller than 15 mm showed typical finding of HCC less frequently than HCCs of 15 mm or larger in diameter. In subgroup analyses, HCCs with diameters between 10 and 15 mm showed similar MRI findings to HCCs with diameters of 10 mm or less but significantly different findings compared with HCCs with diameters from 15 to 20 mm and 20-30 mm. Conversely to Choi we found that in our series (tumor with diameter between 10-20 mm), the typical hallmark without the cut-off of 15 mm. So we think, in accordance to Becker [26], that the presence of wash-out is a crucial step wise LI-RADS decision tree for LR3-5 observations.

Capsule presence is identified as a peripheral rim of smooth hyperenhancement in the portal or delayed phase. The enhancement rim is not always a true tumor capsule, but may represent a pseudocapsule conforming to fibrous tissue and dilated sinusoids around a nodule [22–23]. In our series capsule appearance was present in 111 (89,5%) HCCs, with sensitivity of 87,40%, specificity of 100%, PPV of 100% and NPV of 60,0% and a diagnostic accuracy of 89,40%. We no found difference between the two different contrast media, conversely to Dioguardi Burgio [28]. Our data were similar to that reported by Anis that showed as the capsule appearance has a high positive predictive value for patients at risk of HCC [29].

Threshold growth is defined as a diameter increase (a minimum of 5 mm and a sufficient rate). The necessary growth rate is either at least a 50% increase in diameter compared with baseline within 6 months or at least a 100% increase in diameter over more than 6 months [1–2]. In our series no HCCs showed a threshold growth while we found in a single case that a nodule at 6 months become hypervascular on arterial phase (at first MR examination it was detected only by hepatospecific phase). So we think that during the follow-up it should be considered not only the threshold growth but also the appearance of imaging features that before were not existing as hypervascular feature.

All HCCs in this study were hypointense on hepatospecific phase of contrast study, with sensitivity of 100,00%, specificity of 70,83%, PPV of 94,78, NPV of 100,00 and a diagnostic accuracy of 95,36. Our results are in agreement with those reported by others [30–36]. According to Golfieri et al, during the hepatospecific phase, typical HCC and early HCC appear hypointense, while low-grade dysplastic or regenerative nodules appear as iso- or hyperintense lesions. EOB-MRI diagnostic accuracy to early HCC diagnosis was approximately 95,00-100,00% [33]. One third of hypovascular hypointense nodules in hepatospecific phase become hypervascular progressed HCC, with a 1 and 3-year. Therefore, the authors suggested that these hypovascular nodules should be rigorously followed up or treated as typical HCC [33]. In the study by Ahn et al. [35], 9 out of 84 HCCs (10.7%) were exclusively identified by hepatospecific phase and three were early HCCs, while in Golfieri et al [34] 19 out of 20 early HCC remained unclassified at dynamic MRI alone because of atypical behavior and were diagnosed only in the hepatospecific phase [34]. In this study 3 out of 15 nodules detected by hepatospecific phase were HCC and one became hypervascular progressed HCC, according to Golfieri [33]. So we are in agreement with Golfieri et al [36] that suggested that in atypical cirrhotic nodule, the hepatospecific phase hypointensity is the most pertinent diagnostic symbol to discriminate low-risk and high-risk nodules, since the reduction of Gd-EOB-DTPA uptake appears to occur at an early stage of hepatocarcinogenesis preceding with the portal blood flow reduction and nodule arterialization [37]. So that in hypervascular atypical HCC hepatospecific phase hypointensity can be used as the second malignancy sign. Therefore, we think that the hypointensity on hepatospecific phase should be considered as a major features in LI-RADS.

According to LI-RADS, T2-W hyperintensity is an ancillary imaging features. Previous study demonstrated that T2-W hyperintensity was a highly precise indicator of nodule malignancy, although poorly sensitive [38–40], while in Golfieri et al T2-W hyperintensity was a poor predictor of malignancy in the early stages of HCC [36]. Conversely to Golfieri [36], Ouedraogo et al [41] demonstrated that the addition of T2-W hyperintensity to the AASLD criteria increased the detection rate of HCC, especially nodules smaller than 20mm, increased the sensitivity of MRI from 67.6 % to 79 %. In our series 107 (84,2%) out of 127 HCCs were hyperintense on T2-W sequences; all typical nodules were hyperintense on T2-W while 57,5 % out of atypical nodules were hyperintense on T2-W, suggesting that there was a correlation between arterialization and signal intensity on T2-W. T2-W hyperintensity showed sensitivity of 84,25, specificity of 100,00, PPV of 100,00, NPV of 54,55 and a diagnostic accuracy of 86,75. In our series all lesions T2-W hyperintensity showed restricted diffusion with hypointense signal on ADC map (107 out of 127). So DWI showed the same sensitivity, specificity, PPV, NPV and diagnostic accuracy than T2-W. The role of DWI in HCC patient has been evaluated by different studies [42–48]. Lee et al demonstrated that the added use of DWI to the MRI with gadoxetic acid-enhanced could be a guideline to discriminate HCCs and dysplastic nodules. In their study, 86 HCCs (84.3%) showed hyperintensity on DWI, whereas only three dysplastic nodules (13.0%) showed this feature. So they concluded that DWI hyperintensity was extremely indicative of HCC in patients with chronic hepatitis or cirrhosis [44]. Also Piana et al [45] showed that arterial phase enhancement and DWI hyperintensity were more sensitive criteria for HCC compared to conventional criteria (77–76% versus 60% for all HCCs and 66–60% versus 37% for HCCs smaller than 20 mm). Sensitivity obtained using the arterial-dominant phase enhancement and washout (in the portal venous and/or equilibrium phases) or hyperintensity on DWI was higher (84–85% for all HCCs and 71–74% for HCCs smaller than 20 mm). In our previous study we demonstrated that that DWI could be used to predict the histological grade of HCC; in fact we found that there was a good correlation between ADC and grading, between fp and grading, and between Dt and grading [46]. Nakanishi et al [47] showed not only the usefulness of DWI for histological grading, but also the possibility to use ADC as a preoperative prediction of early HCC recurrence within 6 months of operation. Conversely, Nasu et al [48], in a series of 125 resected HCCs (sizes range: 0.8–15 cm), found no correlation between histological grade and ADC (using b factors of 0 and 500 s/mm^2^), although the DWI and Signal Intensity of the HCCs increased in higher grade. We found that the median value for ADC was 1,47 x10^-3^ mm^2^/s (range 0,94-2,44 x10^-3^mm^2^/s), for fp was 33,3% (range 12,14-54,08%), for Dp was 45,3 x10^-3^ mm^2^/s (range 13,7-52,7 x10^-3^ mm^2^/s) and for Dt was 0.9×10^-3^ mm^2^/s (range 0.81-1,51 x10^-3^mm^2^/s) with an overlapping of ADC and IVIM parameters values among typical and atypical nodules. There was not difference significant statistically between typical and atypical nodules using ADC or IVIM parameters values (p value > 0.05 at Kruskal Wallis test). These results suggested that there was not a correlation between ADC or IVIM parameters values and hypervascularization during arterial phase.

In this study we employed a 1.5T MR scanner, however we think that our results could not change with use of a 3T scanner. In fact, although 3T systems are advantageous for musculoskeletal, neuroimaging, and angiographic applications few articles have been published regarding their use for abdominal and, particularly, liver examinations [49–50]. The quality of 3T images on liver studies is reported to be equivalent to 1.5T images [49], this in fact depends on the individual sequences and the particular machine [50]. Moreover, a drawback of 3T is an increased number of types of artefacts. Certain imaging artefacts are more prominent at 3T than at 1.5T, mainly because their physical parameters are dependent on the main magnetic field strength (B0): chemical shift artefacts of the first kind are directly proportional to the B0 and generally are twice as prominent with 3T imaging, noticeable on in-phase T1-weighted images; susceptibility artefacts also increase with the B0 and are approximately twice as prominent at 3T as compared with 1.5T.

## MATERIALS AND METHODS

### Patient population

A retrospective study, approved by National Cancer Institute Pascale Foundation of Naples, was performed through a computerized search of medical records on 160 patients underwent liver MR imaging and followed by biopsy for HCC from August 2010 to February 2017. After reviewing the medical records, 52 patients were excluded because the tumors were bigger than 2 cm; 34 patients because the tumors were smaller than 1 cm and 4 were excluded because the final pathology report was confirmed not to be HCC. The final study population included 70 patients (33 women and 37 men; mean age 68 years; range: 52-83 years) with 173 nodules (all with tumor diameter between 1 to 2 cm). All patients had chronic liver disease which was related to hepatitis C virus infection in 29 patients, hepatitis B virus in 39 cases, and alcohol abuse in 2; all were stage A according to the Barcelona-Clinic Liver Cancer (BCLC) classification; alpha-fetoprotein levels were >4 ng/ml (12-320 ng/ml, mean 80 ng/ml) in all patients. All patients underwent MR study at 0 time (MR0), after 3 (MR3) and 6 months (MR6) using two different contrast media (CM). Liver-specific agent, taken up by hepatocytes (Gd-EOB-BTPA), was injected at MR0 and MR6, non-specific cm that distributes into the vascular and extravascular extracellular spaces (Gd-BT-DO3A) was injected at MR3. The mean interval standard deviation between pathologic examination and last MR study (MR6) was 15 days (range 4–28 days).

### MR imaging protocol

MR imaging was performed by using a 1.5T MR (Magnetom Symphony, with Total Imaging Matrix Package, Siemens, Erlangen, Germany) with 8-element body and phased array coils. The MRI examination consisted of basal images taken before IV administration of contrast medium and then functional dynamic sequences obtained after IV injection of cm, acquiring the last series of images, when we used hepatospecific cm, with a delay of 20 minutes during the hepatobiliary excretion of the cm. The baseline sequences obtained before IV contrast medium were coronal True fast imaging with steady state precession (TrueFISP) T2-weighted free breathing; axial Half-Fourier Acquisition Single-Shot Turbo Spin-Echo (HASTE) T2-weighted, with controlled respiration, without and with fat-suppressed (FS) gradient-echo pulse; coronal HASTE T2-weighted, without FS; axial flash in-out phase T1-weighted, with controlled respiration; Volumetric Interpolated Breath-hold Examination (VIBE) T1-weighted SPAIR with controlled respiration; diffusion weighted imaging (DWI) with planar echo-pulse sequence (EPI) at several b value *b* value 0, 50, 100, 200, 400, 600, and 800 s/mm^2^. As liver-specific CM, the EOB-Gd-BPTA (Primovist, Bayer Schering Pharma, Germany) was employed. All patients received 0.1 ml/kg of EOB-Gd-BPTA by means of a power injector (Spectris Solaris® EP MR, MEDRAD Inc., Indianola, IA, USA), at an infusion rate of 1 ml/s. As non-specific agent the Gd-BT-DO3A (Gadovist, Bayer Schering Pharma, Germany) was employed. All patients received 0.1 ml/kg of Gd-BT-DO3A by means of a power injector (Spectris Solaris® EP MR, MEDRAD Inc., Indianola, IA, USA), at an infusion rate of 2 ml/s. After contrast medium administration, VIBE T1-weighted FS (SPAIR) sequences were acquired in different phases: hepatic arterial (35 s delay), portal venous (90 s), equilibrium (120 s), and hepatobiliary excretion (20 minutes). Details of sequence parameters were reported in Table 4.

**Table 4:** Pulse Sequence Parameters on MR studies

Sequence	Orientation	TR/TE/FA(ms/ms/deg.)	AT(min.)	Acquisition Matrix	Slice thickness/Gap (mm)	Fat Suppression
TrueFISPT2-W	Coronal	4.30/2.15/80	0.46	512×512	4/0	without
HASTE T2-W	Axial	1500/90/170	0.36	320×320	5/0	Without and with (SPAIR)
HASTE T2w	Coronal	1500/92/170	0.38	320×320	5/0	without
In-Out phase T1-W	Axial	160/2.35/70	0.33	256×192	5/0	without
DWI	Axial	7500/91/90	7	192×192	3/0	without
VIBET1-W	Axial	4.80/1.76/12	0.18	320×260	3/0	with (SPAIR)

Abbreviations: W= Weighted, TR = Repetition time, TE = Echo time, FA = Flip angle, AT = Acquisition time, SPAIR = Spectral Adiabatic Inversion Recovery, TrueFISP = True fast imaging with steady state precession, HASTE = Half-Fourier acquisition single-shot turbo spin-echo, DWI = Diffusion-weighted imaging, VIBE = Volumetric interpolated breath hold examination.

### Images analysis

Three expert hepatic radiologists retrospectively and independently reviewed all images and served as the consensus. The observers were blinded to clinical history and previous imaging studies. We analyzed only lesion smaller than 2 cm and greater than 1 cm. We used LI-RADS v2014 classification and histological analysis served as the standard of reference.

For each nodule we recorded, according to LI-RADS classification, the size, the presence and quality of arterial-phase hyperenhancement, washout appearance, capsule appearance, threshold growth. Additionally, we recorded signal intensity (SI) on T2-W images, on DWI, on maps of apparent diffusion coefficient (ADC) and, when we used EOB-GD-BPTA, SI on T1-W images during hepatospecific phase on contrast study. For each nodule, we reported the median ADC value and median values of Intravoxel incoherent motion (IVIM) related parameters of pseudo-diffusivity (Dp), perfusion fraction (fp), and tissue diffusivity (Dt).

When we found arterial-phase hyperenhancement, washout and capsule appearance, we defined the HCC as typical. When we found, washout and capsule appearance, but not the arterial-phase hyperenhancement, we defined the HCC as atypical.

The lesion was categorized hyperintense, isointense or hypointense relative to the surrounding liver parenchyma. The lesions that were seen only as hypointense on the hepatobiliary phase were graded as LI-RADS 3.

For lesion detection with DWI, the observers analyzed all b values.

Each observer independently evaluated the presence of arterial phase hyperenhancement using a four-point scale (1 = absent, 2 = low intensity, 3 = mild intensity, 4= high intensity), to compare the efficacy of the two different contrast media to evaluate hyperenhancement.

Also, each observer independently evaluated the degree of image quality degradation caused by respiratory ghost, pulsatile blood flow ghost, and susceptibility artifacts using a four-point scale (1 = absent or minimal, 2 = mild, 3 = moderate, 4 = severe). A “severe” score indicated that an image was uninterpretable and a “mild” score indicated that the artifact did not affect interpretation.

A consensus read was performed when there was disagreement between the readers.

In this study the diffusion parameters estimation was performed using the intravoxel incoherent motion method [6–7].

Bi-exponential model to estimate the IVIM-related parameters of pseudo-diffusivity (D_p_), perfusion fraction (f_p_), and tissue diffusivity (D_t_) was described by the following equation

$$\frac{S_{0}}{S_{b}}={\text{f}}_{\text{p}.}\exp\left(-\text{b }\cdot \;{\text{D}}_{\text{p}}\right)+\left(1-{\text{f}}_{\text{p}}\right)\cdot \exp\left(-b\cdot D_{t}\right)\;$$

We used a VARiable PROjection approach to estimate the three parameters because the bi-exponential model may often be ill-conditioned because of a limited number of samples, small perfusion fraction and/or similar compartmental diffusivities. In a previous study, we have demonstrated that the VARiable PROjection algorithm is superior to the conventional Levenberg–Marquardt algorithm for non linear curve fitting in intravoxel incoherent motion method for DW-MRI data analysis [8]. A brief explanation of VARPRO approach is described in the following.

Rearranging the equation (1) the $S(b)/S_{0}-e^{-bD_{t}}$ is the product of f and a nonlinear function of Dt and Dp:

$${\text{f }(\text{D}}_{\text{p}}{;\text{D}}_{\text{t}};\text{ b})\;=S(b)/S_{0}-e^{-bD_{t}}=f(e^{-bD_{p}}-e^{-bD_{t}})$$

Letting ${\text{f }(\text{D}}_{\text{p}}{;\text{D}}_{\text{t}};\text{ b})\;$ the cost functional becomes:

$$S(b)/S_{0}.-e^{-bD_{t}}={\text{||y-f }(\text{D}}_{\text{p}}{;\text{D}}_{\text{t}}{;\text{ b})\text{ f}||}_{2}\;$$

Therefore, a separable nonlinear least square model known as VARiable PROjection (VARPRO) can be used to calculate the diffusion parameters. If we knew, the estimate of the nonlinear parameters Dp and Dt the estimate of the linear parameter f could be obtained by:

$$f=e^{-bD_{t}}+\text{y}+{\text{f }(\text{D}}_{\text{p}}{;\text{D}}_{\text{t}}{;\text{ b})}^{+}$$

where ${\text{f }(\text{D}}_{\text{p}}{;\text{D}}_{\text{t}}{;\text{ b})}^{+}$ is the Moore-Penrose generalized inverse of ${\text{f }(\text{D}}_{\text{p}}{;\text{D}}_{\text{t}};\text{ b})$. Therefore, a new cost functional can be constructed:

$$S(b)/S_{0}.-e^{-bD_{t}}={\text{||y-f }(\text{D}}_{\text{p}}{;\text{D}}_{\text{t}}{\text{; b) f }(\text{D}}_{\text{p}}{;\text{D}}_{\text{t}}{;\text{ b})}^{+}{\text{y||}}_{2}$$

This analysis was ROI-based using median value of single voxel signals for each b value. ROIs for the tumor were manually drawn to include such hyperintense voxels on image at b value 800 s/mm^2^. No motion correction algorithm was used but ROIs were drawn taking care to exclude areas in which movement artifacts or blurring caused voxel misalignments.

The data analysis was performed using an in-house software written in Matlab (The MathWorks, Inc., Natick, MA, USA).

### Statistical analysis

Data were expressed in terms of median value ± range. Kruskal Wallis non-parametric test was performed to emphasize significant statistically difference between median value in different population subgroups. Sensitivity, specificity, positive predictive value (PPV), negative predictive value (NPV) and accuracy were assessed. Fisher's exact test was used to evaluate statistical significance of dichotomous 2×2 tables. A p value < 0.05 was considered statistically significant. All analyses were performed using Statistics Toolbox of Matlab R2007a (The Math-Works Inc., Natick, MA).

## CONCLUSION

LI-RADS is a system score to interpret and report imaging features in patients at risk for HCC. Although the arterial phase hyperenhancement is an essential prerequisite for definitely HCC, it not sufficient for LR-5 categorization. Moreover the degree of hyperenhancement is higher with Gd-BT-DO3A than GD-EOB-DTPA, so as the image quality degradation was lower with Gd-BT-DO3A compared to GD-EOB-DTPA. However considering that the hyperenhancement during arterial phase has a lower sensitivity, specificity, PPV, NPV and diagnostic accuracy than hypointensity on hepatospecific phase and that hypointensity in the hepatospecific phase and wash-out appearance are the most significant diagnostic signs to discriminate low-risk from high-risk nodules, patients at risk for HCC may be evaluated alternating these contrast media. The capsule appearance, T2-W hyperintensity and restricted diffusion have a high positive predictive value for HCC while threshold growth may be associated to other features as the appearance of arterial phase hyperenhancement.
